# Biosynthesis of Silver Nanoparticles from *Duchesnea indica* Extracts Using Different Solvents and Their Antibacterial Activity

**DOI:** 10.3390/microorganisms11061539

**Published:** 2023-06-09

**Authors:** Se-Min Kim, Hye-Jo Choi, Jeong-A Lim, Min-Ah Woo, Hyun-Joo Chang, Nari Lee, Min-Cheol Lim

**Affiliations:** 1 Research Group of Food Safety and Distribution, Korea Food Research Institute (KFRI), Wanju 55365, Republic of Korea; 2 Department of Food Science and Technology, Jeonbuk National University, Jeonju 54896, Republic of Korea; 3 Department of Food Biotechnology, Korea University of Science and Technology, Daejeon 34113, Republic of Korea

**Keywords:** biosynthesis, green synthesis, silver nanoparticles, plant extracts, *Duchesnea indica*

## Abstract

Silver nanoparticles (AgNPs) were synthesized using the whole plant of *Duchesnea indica* (DI) which was extracted in different solvents; the antimicrobial effects of the extract were investigated in this study. The extraction of DI was performed using three different solvents: water, pure ethanol (EtOH), and pure dimethyl sulfoxide (DMSO). AgNP formation was monitored by measuring the UV–Vis spectrum of each reaction solution. After synthesis for 48 h, the AgNPs were collected and the negative surface charge and size distribution of the synthesized AgNPs were measured using dynamic light scattering (DLS). The AgNP structure was determined by high-resolution powder X-ray diffraction (XRD) and the AgNP morphology was investigated using transmission electron microscopy (TEM). AgNP antibacterial activities were evaluated against *Bacillus cereus*, *Staphylococcus aureus*, *Escherichia coli*, *Salmonella enteritidis*, and *Pseudomonas aeruginosa* using the disc diffusion method. Additionally, minimum inhibitory concentration (MIC) and minimum bactericidal concentration (MBC) values were also determined. Biosynthesized AgNPs showed enhanced antibacterial activity against *B. cereus*, *S. aureus*, *E. coli*, *S. enteritidis*, and *P. aeruginosa* compared with that of pristine solvent extract. These results suggest that AgNPs synthesized from extracts of DI are promising antibacterial agents against pathogenic bacteria and can be further applied in the food industry.

## 1. Introduction

Food poisoning incidents continue to occur frequently despite significant advancements in science and technology. Given the challenges associated with preventing food poisoning outbreaks, it becomes crucial to focus on both preventing infections caused by bacteria and viruses and effectively managing contamination through rapid detection methods. Particularly, fresh food products, due to limited pretreatment processes such as heating, are susceptible to bacterial or viral contamination through various routes, leading to an increased risk of food poisoning [1,2,3]. Therefore, there is an urgent need for microbial control technologies during the distribution of fresh food to ensure food safety and prevent potential outbreaks. Accordingly, various studies on food packaging with antibacterial properties are being conducted. In particular, approaches using nanotechnology in packaging materials are attracting attention [4]. Nanotechnology is being researched in various fields, such as medicine, chemistry, and food packaging materials. Among these, silver nanoparticles (AgNPs) are the most widely commercialized materials [5]. Recently, AgNPs were used in relation to antibacterial, antifungal, and antiviral agents, and are receiving much attention in the field of catalysts [6,7,8,9]. AgNPs have a large surface area, small size, high dispersion, and antibacterial effects but show little toxicity in human cells [10,11,12,13,14]. Based on these characteristics, the applicability of AgNPs as food packaging materials has been suggested [15,16,17].

AgNP synthesis is normally categorized into physical, chemical, or biological methods [18,19,20,21]. Among these approaches, biological synthesis methods using bacteria, fungi, yeast, and plants do not require toxic chemicals for production and do not produce harmful by-products. In biological synthesis, plant-based methods are more ecofriendly and cost-effective than other methods. Plants contain various biomolecules, such as flavonoids and phenols, that facilitate rapid silver ion bioreduction, which is advantageous for AgNP production. Therefore, AgNP production using various plants and the characteristics of the resulting AgNPs have been investigated in previous studies [22,23,24,25].

*Duchesnea indica* (DI) is a perennial plant belonging to the Rosaceae family that is widely distributed in South and East Asia. Since ancient times, DI fruit has been used in the treatment of stomach pain and high fever, while the plant has been used in medicine to treat cancer and intestinal poisoning. DI contains various phytochemicals, including flavonoids, sterols, triterpenes, polyphenols, and volatile oils. Therefore, DI is a good candidate species for synthesizing AgNPs [26,27]. Ihsan et al. extracted phytochemicals from DI roots using a water-soluble solvent and investigated AgNP synthesis [28]. In another study, solvents with different polarities were evaluated for their effect on the antibacterial activities of AgNPs synthesized using *Cinnamomum zeylanicum* [29]. Although previous studies showed that DI root extracts collected using water as a solvent can form biologically active AgNPs, there are no reports on AgNP biosynthesis from DI plant extracts isolated using different solvents as a biomolecule source.

In this study, three types of DI extracts were obtained using different solvents: water, pure ethanol (EtOH), and pure dimethyl sulfoxide (DMSO). Subsequently, AgNPs were synthesized using the collected extracts under controlled reduction conditions. The generated AgNPs were characterized using UV–Vis spectroscopy, dynamic light scattering (DLS), zeta-potential, X-ray diffractometry (XRD), and transmission electron microscopy (TEM). The antibacterial activity of each solvent extract and the produced AgNPs were tested against bacteria such as *Bacillus cereus*, *Escherichia coli*, *Salmonella enteritidis*, *Staphylococcus aureus*, and *Pseudomonas aeruginosa*.

## 2. Materials and Methods

### 2.1. Materials

DI plants were purchased from a local market in Jeonju, Republic of Korea. Silver nitrate, ethanol, and DMSO were purchased from Sigma-Aldrich (St. Louis, MO, USA). Tryptic soy broth (TSB) and Muller Hinton broth (MHB) were acquired from BD Biosciences (Sparks, MD, USA). All the working solutions were prepared in deionized water.

### 2.2. Preparation of Duchesnea Indica Extracts

DI was washed several times with running water to remove impurities. The plants were then dried and ground into powder before extraction. Powdered DI (3 g) was mixed into 60 mL of each extraction solvent (water, ethanol, or DMSO) for 48 h. The extraction temperatures were 80 °C for water and 20 °C for ethanol and DMSO. The crude extracts were filtered using Whatman No. 1 filter paper (Whatman Ltd., Maidstone, UK) and the filtrate was stored at 4 °C. Subsequently, *D. indica* extracts were used as reducing and stabilizing agents during AgNP synthesis.

### 2.3. AgNP Biosynthesis and Characterization

To synthesize AgNPs, 4 mL of each DI extract was added to 16 mL of 1 mM silver nitrate solution. The mixture was allowed to react at room temperature for 24 h. AgNP formation was monitored by measuring the UV–Vis spectrum of each reaction solution using a SpectraMax i3X microplate reader (Molecular Devices, Sunnyvale, CA, USA) from 300–700 nm. The formed AgNPs were collected by centrifugation at 3000× *g* for 20 min and were washed with DW using a 10 kDa weight cut-off filter (Amicon Ultra-15 MWCO, Merck Millipore, Burlington, MA, USA). The obtained AgNPs were resuspended in 4 mL of DW. The surface charge and size distribution of the synthesized AgNPs were measured by DLS using a Zetasizer Nano ZS90 instrument (Malvern Instruments, Malvern, Worcestershire, UK). The structure of the synthesized AgNPs was determined by high-resolution powder XRD using an Empyrean instrument (PANalytical, Almelo, The Netherlands) in the 10°–80° 2*θ* range with Cu Kα radiation. The AgNP morphology was determined by TEM using an H-7650 electron microscope (Hitachi, Tokyo, Japan). Each sample was drop-casted onto a carbon 200 mesh copper grid (Ted Pella, Redding, CA, USA) and dried in a vacuum desiccator overnight.

### 2.4. Antibacterial Activity

The antibacterial activity of DI extracts prepared using different solvents and AgNPs synthesized from each extract was determined using the disc diffusion method. *B. cereus* (ATCC 14579), *S. aureus* (ATCC 25923), *S. enteritidis* (ATCC 13076), *E. coli* (ATCC 10536), and *P. aeruginosa* (ATCC 15692) were used in all experiments. All bacteria were inoculated in TSB and incubated overnight at 37 °C. The bacterial suspensions were adjusted to 10^6^ CFU/mL and spread on MHA plates using sterilized cotton swabs. To examine the antibacterial effect of AgNPs according to the solvent, DI extracts were prepared using different solvents, AgNPs were synthesized from each extract, and AgNO_3_ (1 mM) was compared. As a positive control, kanamycin (10 µg) was used as a standard antibiotic and gentamicin sulfate (10 µg) was used only for *P. aeruginosa*. Each sample (100 µL) was impregnated into a sterilized cellulose disk (∅8 mm, Adventec, Tokyo, Japan) and the dried discs were placed on the plate inoculated with bacteria. All plates were incubated at 37 °C for 24 h and the diameter of the inhibition zone was measured.

### 2.5. Determination of Minimum Inhibitory Concentration (MIC) and Minimum Bactericidal Concentration (MBC)

The MIC and MBC of *D. indica* extract and AgNPs were determined against *B. cereus*, *S. aureus*, *S. enteritidis*, *E. coli*, and *P. aeruginosa* strains using the macro broth dilution method. Bacteria strains were inoculated in Muller Hinton broth (MHB) and incubated overnight at 37 °C. DI extract and AgNPs were serially diluted two-fold and 100 µL was added into each tube containing 900 µL of MHB. The final concentration of DI extract and AgNPs ranged from 0.32 to 5 mg/mL and 0.06 to 1 mg/mL, respectively. Then, 10 µL of the bacterial suspension was inoculated into each tube to contain 10^6^ CFU/mL. Positive (medium containing bacteria without DI extracts or AgNPs) and negative (medium containing DI extract or AgNPs without bacteria) control tubes were also prepared. All tubes were incubated at 37 °C for 24 h. MIC was defined as the lowest concentration that inhibited the visual growth of test cultures. After that, 100 µL from all tubes without turbidity (with concentrations ≥ MIC) were subcultured on MHA plates and incubated at 37 °C for 24 h. MBC was determined as the lowest concentration at which no bacterial growth was observed.

## 3. Results and Discussion

### 3.1. Biosynthesis of AgNPs Using Different Solvent-Assisted Extracts

**Figure 1:**
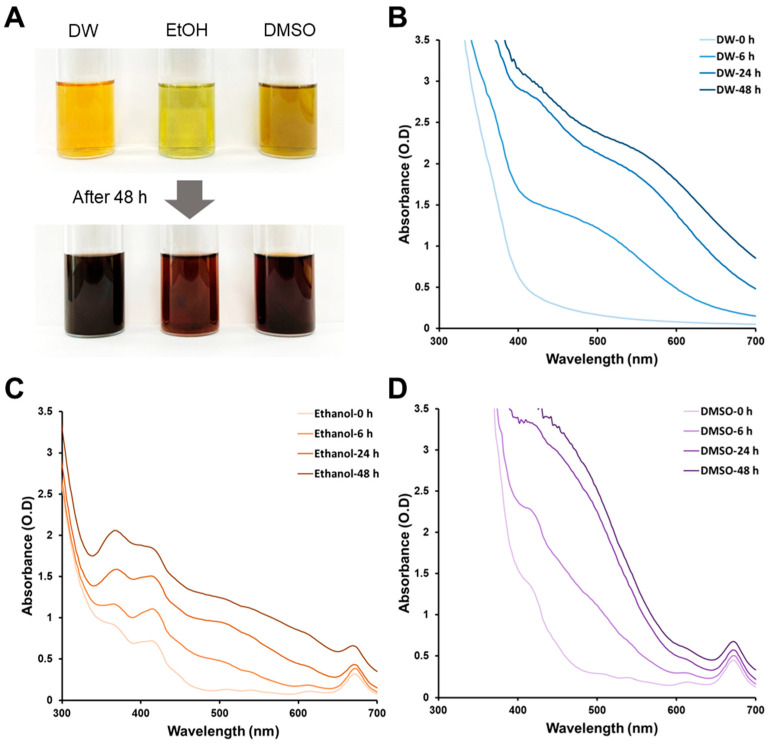
Color changes of different extracts after silver nanoparticle formation (**A**). UV–Vis spectra of silver nanoparticles synthesized using distilled water (DW) extract (**B**), ethanol extract (**C**), and DMSO extract (**D**) over time.

We used silver ions that were reduced by DI extracts in different solvents to form colloidal AgNPs. Figure 1 shows the effect of the extraction solvents DW, ethanol, and DMSO on AgNP biosynthesis. Highly polar water, highly polar organic DMSO, and moderately polar organic ethanol were used to extract various phytochemicals from plant samples. The extracted color varied depending on the solvent used, indicating differences in the nature of the biomolecules contained in each extract [30,31,32]. AgNP biosynthesis was visually observed by the change in the reaction solution color after a reduction time of 48 h (Figure 1A). For water extracts, the color change occurred most rapidly after 6 h, while the ethanol extract showed the slowest AgNP biosynthesis (Figure 1B–D). The multiple peaks for ethanol and DMSO were caused by the extracted biomolecules and increased by the formation of AgNPs throughout the wavelength range. The reduction in silver ions using plant extracts may be affected by solvents with different polarities [29]. Color changes in the reaction solution containing DMSO extract were the least after 24 h, while the color of the ethanol extract gradually increased in absorbance over time. The influence of solvents on AgNP biosynthesis was investigated in this result. It was observed that the extraction solvents played a significant role in the synthesis process. The color changes in the reaction solutions, indicating AgNP formation, varied depending on the solvent used. Therefore, the different polarities of solvents are believed to influence the reduction in silver ions using plant extracts. These results indicate that the biomolecule content and concentration affect AgNP biosynthesis. 

**Figure 2:**
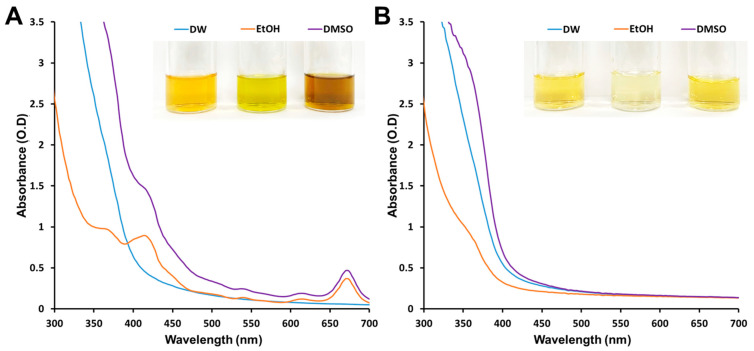
UV–Vis spectra of solutions containing different solvent extracts before reaction (**A**) and filtered solutions after 48 h reaction and silver nanoparticle collection using a molecular weight cut-off filter (**B**). The insets are representative images of solutions pre-reaction (**A**) and post-filtration (**B**).

The diversity and number of biomolecules in the solvent were evaluated using absorbance measurements. Purified extracts were diluted in an aqueous solution (the same volume as that of the reduction process). The molecular complexity of the DW-based extract was the lowest among the tested extraction samples, while the ethanol and DMSO samples showed clear absorbance peaks at approximately 410 nm and 670 nm, respectively (Figure 2A). After 48 h, the biosynthesized AgNPs were collected using an MWCO filter for subsequent characterization and antimicrobial testing. As shown in the Figure 2B inset, the eluted reaction solutions which passed through the MWCO filter had a pale color compared to the pre-reaction solution. The color change in the reaction solution caused by AgNP synthesis was most clearly observed in the DMSO sample. For the DMSO extract, the solution color changed from brown to pale yellow. For the ethanol extract, the olive-colored solution became nearly transparent. The distinct absorbance peaks disappeared after reduction by the ethanol and DMSO samples for 48 h (Figure 2B). These results indicate that the biomolecules contained in the extraction solution act as reducing agents or stabilizers during AgNP synthesis. In particular, the biomolecules contained in the ethanol and DMSO extracts have a greater effect on the synthesized AgNPs than those in the water extract.

### 3.2. Characterization of Biosynthesized AgNPs

**Figure 3:**
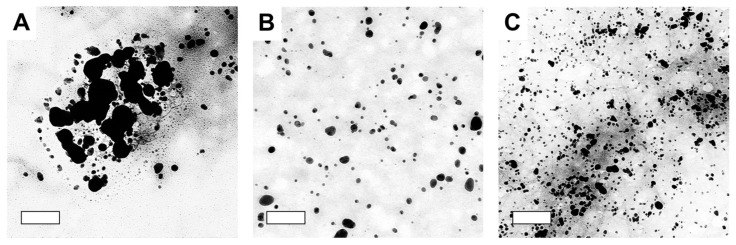
Transmission electron microscopy (TEM) images of silver nanoparticles synthesized using different extraction solvents. (**A**) Distilled water extract, (**B**) ethanol extract, and (**C**) DMSO extract. The scale bar in TEM images is 200 nm.

Morphological characterization of the synthesized AgNPs was conducted by TEM analysis. Figure 3 shows TEM images confirming the morphology and size of the metalized NPs synthesized using different extraction solvents. The AgNPs produced by water extracts were mainly irregular in shape owing to the aggregation of adjacent AgNPs, which was likely caused by the absence of capping molecules [33]. These reaction conditions possibly influenced the formation of larger AgNPs in the aqueous extract. The AgNPs synthesized using ethanol and DMSO extracts were mainly spherical with different sizes and size distributions. This result was due to the difference in the AgNP synthesis rate and the difference in the composition of the biomolecules.

The zeta potentials of all the AgNPs are shown in Table 1. The zeta potentials of AgNPs synthesized using DW, ethanol, and DMSO extracts were −22.0 ± 0.3, −19.3 ± 0.2, and −19.0 ± 0.6, respectively. The absolute value of the surface charge of the biosynthesized AgNPs is inversely proportional to the amount of biomaterial adsorbed on the particle surface [34]. The zeta potential data suggest that AgNPs synthesized using DMSO extract were more densely capped by polyphenolic compounds than those synthesized using DW extract. The surface charge of the synthesized AgNPs was also confirmed in relation to the biomolecule diversity and concentration in each extract, as confirmed by the absorbance spectra in Figure 2A. Furthermore, the surface of AgNPs naturally maintains a negative charge due to the interaction of Ag ions with negatively charged ligands during AgNPs formation. Consequently, AgNPs can exhibit negative zeta potentials due to the presence of negatively charged ligands that adsorb onto the nanoparticle surface. This interaction between the ligands and the nanoparticle surface contributes to the maintenance of a negative surface charge, which is reflected in the observed negative zeta potential. This negative zeta potential plays a crucial role in ensuring the stability and dispersion of the AgNPs.

The size distributions of the synthesized AgNPs for each extraction solvent are listed in Table 1. The sizes obtained from DLS analysis were comparable to the TEM images. These results indicate that changing the extraction solvent results in the production of AgNPs of different sizes and surface charges. The morphological properties of AgNPs produced using plant extracts can be modulated by controlling conditions such as the temperature and pH of the reaction solution [35,36]. The surface charge and particle size of synthesized AgNPs significantly influence the colloidal dispersion stability. In the case of AgNPs synthesized from water extracts, although they exhibit a high surface charge, their larger particle size results in lower dispersion stability compared to nanoparticles synthesized from other solvent extracts.

**Figure 4:**
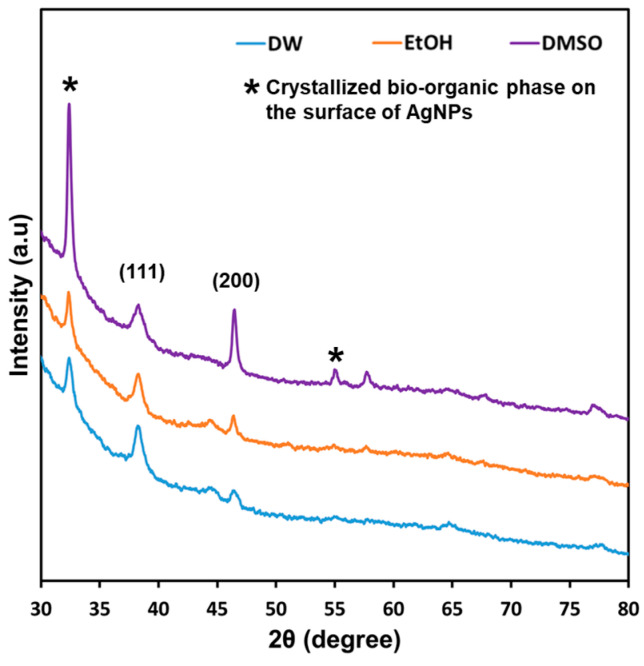
X-ray diffraction pattern of silver nanoparticles synthesized using different extraction solvents. DW, distilled water extract; EtOH, ethanol extract; DMSO, DMSO extract.

Powder XRD analysis was used to characterize the AgNPs synthesized using different extraction solvents to determine the crystal structure and to confirm the presence of AgNPs. In the 2*θ* range of 30–80°, the XRD patterns of the AgNPs confirmed a face-centered crystalline (FCC) structure, as shown in Figure 4. Diffraction peaks were observed at 2*θ* angles of approximately 38° and 44°, which corresponded to the (111) and (200) planes, respectively [37]. The distinct XRD peak patterns for each sample suggest the effectiveness of fabricating AgNPs using *D. indica* extracts. A peak at approximately 33° and 54° (2*θ* angle) suggests the presence of a crystallized bio-organic phase on the surface of the synthesized AgNPs [38]. Additionally, the stronger peaks for AgNPs synthesized using the DMSO-based extract indicate the formation of AgNPs with higher crystallinity and biomolecule complex formation.

### 3.3. Antibacterial Activity of Biosynthesized AgNPs

**Figure 5:**
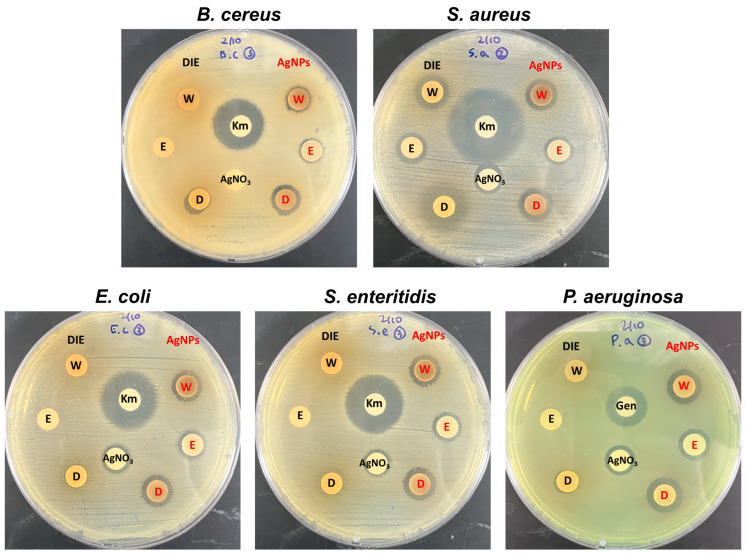
Antibacterial activity results of *Duchesnea indica* extract (DIE) prepared using water (W), ethanol (E), DMSO (D), and synthesized AgNPs. Km, kanamycin; Gen, gentamycin.

The antibacterial activity results of the extract and AgNPs against the target bacteria are shown in Table 2 and representative images of each plate are shown in Figure 5. The *D. indica* (DI) extract showed inherent antibacterial properties depending on the solvent. While the extracts using distilled water (W) and ethanol (E) showed activity only against *S. aureus*, the DMSO-based extract showed activity against all bacteria, with the size of the inhibition zone ranging from 8.52 to 14.92 mm. One interesting aspect observed in our study is the varied antibacterial activity of the DI extract depending on the solvent used for extraction. The DMSO-based extract exhibited broad-spectrum activity against all tested bacteria, which can be attributed to the extraction of different classes of bioactive compounds present in DI. The presence of these bioactive compounds, such as flavonoids, tannins, and phenolic compounds, in the DMSO extract might contribute to its potent antimicrobial effects [26]. This may be related to the concentration and composition of DI-derived biomolecules, as shown in Figure 2A. Variations in the extracted DI phytochemicals based on the solvents used and differences in antibacterial properties were previously reported [26]. Moreover, other studies have reported the application of the ethanol extract of DI as a treatment for sepsis and the water extract of DI as a medicinal cosmetic against skin pathogens such as *S. aureus* and *S. epidermidis* [39,40].

Overall, the synthesized AgNPs exhibited better antibacterial activity than AgNO_3_, but their effect on bacteria varied from that of the DI extract. Gram-negative bacteria were more susceptible to the AgNPs, whereas the DI extract showed higher inhibitory activity against Gram-positive bacteria. *S. aureus*, in particular, showed a different trend than other strains, with the DI extract (11.39–14.92 mm) demonstrating larger inhibition zones than AgNPs (10.82–12.86 mm). The difference in antimicrobial activity between Gram-positive and Gram-negative bacteria is consistent with previous findings. Feng et al. reported that AgNPs show greater antibacterial activity against *E. coli* than *S. aureus*, which was attributed to differences in their cell wall compositions [41]. Gram-positive bacteria have a thick cell wall network composed of peptidoglycan layers that resist mechanical rupture, whereas Gram-negative bacteria have thin cell walls and are more susceptible to metallic nanoparticles [42]. The antibacterial effect of AgNPs also can vary depending on their shape and size, as these factors can affect the surface area and reactivity of the nanoparticles [43]. Smaller nanoparticles are more effective at killing bacteria because they can release a greater amount of silver cations due to their higher surface area-to-volume ratio [44]. The largest zone of inhibition was observed in DMSO-based AgNPs, which could be attributed to the smallest particle size observed in TEM analysis (Figure 3). In addition, the water based-AgNPs observed in TEM may have been affected by severe aggregation which reduces their antibacterial effect. Finding conditions that minimize aggregation have the potential to enhance antibacterial activity of water-based AgNPs. Anzum et al. reported that AgNPs synthesized from an aqueous extract of *Cinnamon zeylanicum* showed superior antibacterial activity compared to DMSO-based AgNPs, which differs from our findings [29]. As a result, AgNPs synthesized from extracts of three solvents (water, EtOH, and DMSO) showed promising antibacterial activity and could be useful as antibacterial agents. Nevertheless, efforts to find ideal synthesis conditions that can exert maximum antibacterial activity are still necessary.

### 3.4. Determination of Minimum Inhibitory Concentration (MIC) and Minimum Bactericidal Concentration (MBC)

The antibacterial efficacy of both plant extracts and synthesized AgNPs can also be expressed as MIC and MBC. Table 3 shows the results of the MIC and MBC of the DI extracts and AgNPs. The water extract did not show activity against all bacteria (>5 mg/mL) and the ethanol extract showed no activity against other bacteria except B. cereus. Among the strains tested, B. cereus had the lowest MIC and MBC values, which ranged from 1.25 to 5 mg/mL for the DMSO extract. The MIC and MBC of all synthesized AgNPs were lower than those of the extracts. EtOH based-AgNPs showed the lowest antibacterial activity, which is consistent with the results of a disc diffusion assay.

One significant finding from our study is the antibacterial effect of the synthesized AgNPs. The MIC and MBC results presented in Table 3 clearly demonstrate the potency of AgNPs against the tested bacteria. The synthesized AgNPs exhibited lower MIC and MBC values compared to the DI extracts, indicating their enhanced antibacterial activity. Notably, the ethanol-based AgNPs showed slightly higher MIC and MBC values than the other AgNP samples, suggesting variations in their antibacterial efficacy depending on the solvent used for synthesis. Furthermore, the MIC and MBC values revealed that S. aureus displayed the highest resistance among the tested strains. This observation aligns with previous studies reporting that S. aureus can exhibit reduced susceptibility to silver-based antimicrobials [10]. However, it is important to note that even though the MIC and MBC values were slightly higher for S. aureus, the synthesized AgNPs still exerted a significant antibacterial effect against this Gram-positive bacterium. These findings reinforce the broad-spectrum antimicrobial activity of AgNPs, which can target both Gram-negative and Gram-positive bacteria, and the ability of AgNPs to effectively inhibit the growth of a wide range of pathogenic bacteria.

## 4. Conclusions

Biosynthesizing AgNPs using plant extracts is an ecofriendly approach for rapid AgNP production. The results of this study demonstrate the effect of the extraction solvent (distilled water, ethanol, and dimethyl sulfoxide) on *Duchesnea indica* extracts used to synthesize AgNPs and the effects on reducing and stabilizing silver ions and AgNPs, respectively. The synthesized AgNPs were characterized using UV–Vis spectroscopy, DLS, zeta potential analysis, XRD, and TEM. AgNPs synthesized from ethanol extract were characterized by their relatively small particle size and low zeta potential. The low zeta potential may be due to the higher AgNPs’ surface coverage by phytochemicals extracted from the whole plant of *Duchesnea indica*. The antibacterial activity of all AgNPs synthesized using the three solvents was shown to effectively inhibit both Gram-negative and Gram-positive bacteria. Based on these results, extracts of DI with distilled water, ethanol, and dimethyl sulfoxide as solvents are suitable for producing AgNPs to control undesired bacteria. Although further studies on the antibacterial mechanism and safety of AgNPs synthesized with the three extracts should be conducted, it is expected that biosynthetic AgNPs using DI extracts can be applied to food packaging materials.

## Figures and Tables

**Table 1:** Zeta potential and size distribution of silver nanoparticles biosynthesized using each extraction solvent.

AgNP Sample	Zeta Potential (mV)	Peak 1 (nm)	Peak 2 (nm)
Distilled water extract	−22.0 ± 0.3	266.9 ± 287.4	21.4 ± 6.4
Ethanol extract	−19.3 ± 0.2	76.4 ± 47.5	9.8 ± 3.6
DMSO extract	−19.0 ± 0.6	78.2 ± 31.2	-

**Table 2:** Antibacterial activity of *Duchesnea indica* extract and biosynthesized AgNPs.

Sample	Bacteria	*B. cereus*	*S. aureus*	*E. coli*	*S. enteritidis*	*P. aeruginosa*
	Solvent	Zone of Inhibition (mm)				
*Duchesnea indica* extract	Water	NA	12.26 ± 0.19	NA	NA	NA
	EtOH	NA	11.39 ± 0.05	NA	NA	NA
	DMSO	9.69 ± 0.23	14.92 ± 0.42	8.52 ± 0.44	8.82 ± 0.04	8.87 ± 0.08
AgNPs	Water	9.30 ± 0.11	11.64 ± 0.32	11.35 ± 0.09	11.20 ± 0.45	12.53 ± 0.13
	EtOH	9.04 ± 0.10	10.82 ± 0.29	10.90 ± 0.13	10.47 ± 0.27	11.57 ± 0.03
	DMSO	10.70 ± 0.22	12.86 ± 0.34	12.20 ± 0.21	11.75 ± 0.29	12.98 ± 0.12
AgNO_3_		8.23 ± 0.25	10.87 ± 0.14	10.12 ± 0.04	10.17 ± 0.39	11.23 ± 0.16
Antibiotics		18.95 ± 0.45	27.88 ± 0.49	22.60 ± 0.76	21.07 ± 0.46	15.96 ± 0.23

NA, No activity. Values represent the mean of three replicates ± SD.

**Table 3:** MIC and MBC results of *Duchesnea indica* extract and biosynthesized AgNPs.

		Bacteria									
		*B. cereus*		*S. aureus*		*S. enteritidis*		*E. coli*		*P. aeruginosa*	
Samples	Solvent	MIC	MBC	MIC	MBC	MIC	MBC	MIC	MBC	MIC	MBC
*Duchesnea indica* extract (mg/mL)	Water	>5	>5	>5	>5	>5	>5	>5	>5	>5	>5
	EtOH	2.5	5	5	>5	>5	>5	>5	>5	>5	>5
	DMSO	1.25	2.5	2.5	5	5	5	2.5	5	2.5	5
AgNPs (mg/mL)	Water	0.5	0.5	1	>1	0.5	0.5	0.5	0.5	0.25	0.5
	EtOH	0.5	1	>1	>1	>1	1	1	1	1	1
	DMSO	0.25	0.25	1	>1	0.25	0.5	0.25	0.5	0.25	0.5

## Data Availability

The data presented in this study are available on request from the corresponding author.
